# Evaluation of the implementation of a clinical pharmacy service on an acute internal medicine ward in Italy

**DOI:** 10.1186/s12913-018-2988-y

**Published:** 2018-04-10

**Authors:** Nicola Lombardi, Li Wei, Maisoon Ghaleb, Enrico Pasut, Silvia Leschiutta, Paolo Rossi, Maria Grazia Troncon

**Affiliations:** 1 Department of Pharmacy, Cambridge University Hospitals NHS Foundation Trust, Hills Road, Cambridge, Cambridgeshire CB2 0QQ UK; 2 Department of Pharmacy Practice and Policy, University College London School of Pharmacy, 29-39 Brunswick Square, Bloomsbury, London, WC1N 1AX UK; 3 School of Life and Medical Science, Department of Pharmacy, Pharmacology & Postgraduate Medicine, University of Hertfordshire, Hatfield, Hertfordshire, AL10 9AB UK; 4 Department of Pharmacy, Azienda Sanitaria Universitaria Integrata di Udine, Via Pozzuolo, 330-33100 Udine, Friuli Venezia Giulia Italy; 5 Division of Internal Medicine, Azienda Sanitaria Universitaria Integrata di Udine, Via Pozzuolo, 330 – 33100 Udine, Friuli Venezia Giulia Italy

**Keywords:** Clinical pharmacist, Elderly, Drug-related problems, Pharmaceutical care, Communication

## Abstract

**Background:**

Successful implementation of clinical pharmacy services is associated with improvement of appropriateness of prescribing. Both high clinical significance of pharmacist interventions and their high acceptance rate mean that potential harm to patients could be avoided. Evidence shows that low acceptance rate of pharmacist interventions can be associated with lack of communication between pharmacists and the rest of the healthcare team. The objective of this study was to evaluate the effect of a structured communication strategy on acceptance rate of interventions made by a clinical pharmacist implementing a ward-based clinical pharmacy service targeting elderly patients at high risk of drug-related problems. Characteristics of interventions made to improve appropriateness of prescribing, their clinical significance and intervention acceptance rate by doctors were recorded.

**Methods:**

A clinical pharmacy intervention study was conducted between September 2013 and December 2013 in an internal medicine ward of a teaching hospital. A trained clinical pharmacist provided pharmaceutical care to 94 patients aged over 70 years. The clinical pharmacist used the following communication and marketing tools to implement the service described: Strengths, Weaknesses, Opportunities and Threats (SWOT) analysis; Specific, Measurable, Achievable, Realistic and Timely (SMART) goals; Awareness, Interest, Desire, Action (AIDA) model.

**Results:**

A total of 740 interventions were made by the clinical pharmacist. The most common drug classes involved in interventions were: antibacterials for systemic use (11.1%) and anti-parkinson drugs (10.8%). The main drug-related problem categories triggering interventions were: no specific problem (15.9%) and prescription writing error (12.0%). A total of 93.2% of interventions were fully accepted by physicians. After assessment by an external panel 63.2% of interventions (96 interventions/ per month) were considered of moderate clinical significance and 23.4% (36 interventions/ per month) of major clinical significance. The most frequent interventions were to educate a healthcare professional (20.4%) and change dose (16.1%).

**Conclusions:**

To our knowledge this is the first study evaluating the effect of a structured communication strategy on acceptance rate of pharmacist interventions. Pharmaceutical care delivered by the clinical pharmacist is likely to have had beneficial outcomes. Clinical pharmacy services like the one described should be implemented widely to increase patient safety.

## Background

Clinical pharmacy is a health science discipline in which pharmacists provide patient care that optimizes medication therapy and promotes health, wellness, and disease prevention [1]. Clinical pharmacy services developed at the end of 1960 in the United Kingdom and United States of America [2, 3] and they are now playing an important role in hospitals aiming to reduce medication errors and Adverse Drug Reactions (ADRs).

Appropriateness of prescribing is defined as the outcome of a process of decision-making that maximises net individual health gains within society’s available resources [4]. Inappropriate medication prescribing in the elderly is rising because of increase of ageing population, number of chronic conditions and number of drugs prescribed. A cohort study has found that 25.8% of elderly receive at least one potentially inappropriate medication [5]. Previous evidence has shown that drug histories taken at admission by a clinical pharmacist and participation of a clinical pharmacist in medical rounds reduce medication errors respectively by 51% and 29% [6]. It has also been shown that clinical pharmacists participating in medical rounds and taking drug histories at admission reduce the number of ADRs [7]. Reduction in medication errors and ADRs following the introduction of clinical pharmacists in hospitals has been shown to have a positive impact on reducing length of hospital stay and mortality rate [8].

A meta-analysis looking at the association of medication use and falling found that the use of sedatives, hypnotics, antidepressants and benzodiazepines had a significant association with falls in elderly patients [9]. Input of the clinical pharmacist in providing information for drug changes that might reduce fall risk has been shown to reduce falls by up to 70% [10, 11].

Acceptance rate of interventions made by clinical pharmacists is key as only those interventions accepted by the healthcare team will have an impact on patient care and might produce cost savings. Ward pharmacy services with a high acceptance rate of interventions made by clinical pharmacists are deemed to be cost-effective by policy-makers. However, evidence shows that acceptance rate of clinical pharmacist interventions can be as low as 53% [12]. Assuming pharmacists with appropriate training and knowledge of pharmacotherapy are employed at ward level a possible cause for the low acceptance rate of pharmacist interventions is lack of communication between the pharmacist and the medical team [12].

There is an assumption that if pharmacists provide evidence base advice on medications, doctors, in turn, will implement them. However, this is not always the case and there is a need to foster active collaboration between the two professions applying models of communication from areas outside healthcare [13].

Several studies report on the introduction of ward based clinical pharmacy services with a satisfactory (> 80%) acceptance rate of interventions made by clinical pharmacists [14–17]. However, description of the reason behind the high acceptance rate of interventions is not reported.

Given there is no evidence on strategies adopted to enhance acceptance rate of interventions made by ward based clinical pharmacists the aim of the study was to assess the effect of a tailored communication strategy on the acceptance rate of interventions made by a clinical pharmacist implementing a ward based clinical pharmacy service targeting patients at high risk of drug-related problems. Characteristics of interventions made are described, their clinical importance is assessed and acceptance rate by doctors is measured.

## Methods

### Setting and patients

The study took place between September 2013 and December 2013 in an acute internal medicine unit of a 1099 beds teaching hospital in Italy. The unit has 39 beds and patients admitted are mostly elderly presenting typically with acute geriatric problems and multiple diseases. The healthcare team looking after patients is made of 5 physicians specialized in internal medicine, nurses, healthcare assistants, 1 physiotherapist, 1 dietician, and 1 social worker.

Patients aged 70 or over admitted to the unit were included in the study. Patients were excluded from the study if they suffered terminal illness (life expectancy < 3 months); if expected length of stay was ≤48 h; if they had been enrolled in the study during a previous admission; and if they had been transferred from another acute unit where they had been cared for by internal medicine physician(s).

### Communication strategy

All key stakeholders (eg. hospital management, pharmacists, doctors, and nurses) were informed on the Strengths, Weaknesses, Opportunities and Threats (SWOT) of the new clinical pharmacy service using a SWOT analysis (Table 1). A SWOT analysis is a technique used in strategic planning to enhance understanding and decision-making in organizations and it can be applied to healthcare organizations when introducing a new service [18].

**Table 1:** SWOT analysis

STRENGTHS	WEAKNESSES
1. Chief pharmacist and Internal Medicine consultant supporting the project.	1. Ward pharmacy service offered only Monday to Friday with no weekend cover.
2. Decrease in medication errors, ADRs and falls enhances patient safety.	2. Night on call ward pharmacy service not provided.
3. Potential for reduction in length of hospital stay and related savings.	3. Potential concerns of doctors and nurses with regard to clinical training of the pharmacist.
OPPORTUNITIES	THREATS
1. Opportunity for the trust to introduce ward clinical pharmacy.	1. Due to the current global financial crisis, it may be difficult to find resources to expand the service in the short term.
2. The trust is a teaching hospital and has therefore the potential for setting up post graduate clinical pharmacy programmes to train staff.	

To prove effectiveness of the new clinical pharmacy service implemented to stakeholders type of interventions made was described, their acceptance rate was measured and their clinical significance was assessed. These goals were Specific, Measurable, Achievable, Realistic and Timely (SMART) in order to guide and facilitate reception of the message by stakeholders [19].

**Fig. 1:**
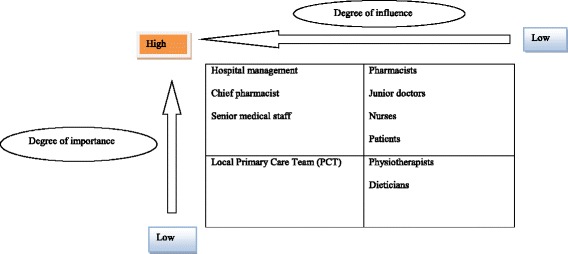
Stakeholders analysis

A marketing strategy targeting key stakeholders according to their degree of influence and importance was developed. Stakeholders’ analysis is reported in Fig. 1.

Hospital managers, chief pharmacist and senior medical staff were targeted with high priority as they would allocate resources and ultimately decide whether to introduce and expand the clinical pharmacy service as part of standard care. Junior doctors, nurses, patients and pharmacists had high importance as they would provide feedback to senior staff and hospital management. However, their influence was low as they were not decision makers. Local PCT had high influence as they were decision makers and were involved in allocating resources.

**Fig. 2:**
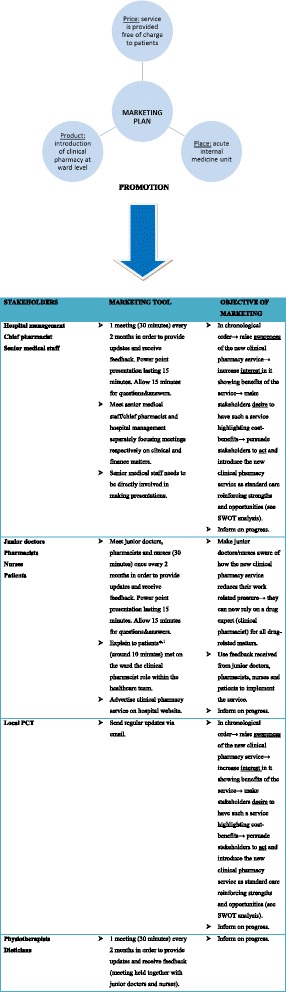
Marketing plan ^*,1^Clinical pharmacist role was not explained to patients unconscious, confused or unable to understand as a consequence of their illness

A marketing plan was developed according to characteristics of the new service implemented and stakeholders (Fig. 2) with particular emphasis on Awareness, Interest, Desire, Action (AIDA model). The AIDA model is used in marketing to help communicate effectively with stakeholders in a way that better responds to their needs and desires [20].

### Intervention

An intervention was defined as any recommendation made by the clinical pharmacist to a healthcare professional, pertaining to drug therapy, which aims to improve the quality of medication use [14]. Recommendations were either made after a prescription was written in order to amend it or before a prescription was written in order to advise on most appropriate prescribing (e.g. statin post stroke in a patient with deranged liver function tests, beta-blocker post myocardial infarction in a patient with severe asthma). The pharmacist covered the internal medicine unit from Monday to Friday spending on average 7 h/day on the unit and providing pharmaceutical care from admission to discharge [21]. The pharmacist had knowledge in geriatric pharmacotherapy and was a trained clinical pharmacist. The pharmacist did ward rounds with the medical team in the morning and follow ups in the afternoon. The main tasks performed were: medicine reconciliation on admission; monitoring and optimization of medicine prescribed during hospital stay; and medicine reconciliation on discharge [22, 23]. Priority was given to new patients and patients going home on the day. The pharmacist contributed to choice of drug regimen, provided evaluated information on pharmaceutical, therapeutic and toxicological aspects of drug therapy, contributed to choice of dosage, form and route of medicine, and helped with dosage calculations [24]. A pharmaceutical care plan was made for each new patient [23, 25]. The pharmacist had access to patient’s medical records and blood tests. Interpretation of drug assays continued to be performed by the clinical pharmacology department as per standard care. When there was an opportunity for optimizing therapy, the pharmacist discussed it with the prescriber who could accept/ partially accept or reject the intervention. The pharmacist answered questions about medications asked by the healthcare team on the ward. Interventions could be triggered either by the pharmacist or by a member of the healthcare team asking a question to the pharmacist. Interventions made were usually communicated orally and recorded afterward in an intervention form. In the event the doctor looking after the patient was not present on the ward interventions made were communicated in writing on a pharmacist intervention note. The note reporting the intervention advised by the pharmacist was attached to the drug chart for the doctor to be reviewed.

### Data collection

Information recorded on the intervention form were: underlying drug-related problem (DRP) category; drug involved (Anatomical Therapeutic Chemical [ATC] code) [26]; description of intervention; outcome; type of intervention and acceptance. Description of the outcome recorded for each intervention reported the changes that had occurred as a direct consequence of the intervention on the day it was made. A DRP was defined as an event or circumstance involving drug therapy that actually or potentially interferes with desired health outcomes [27]. DRPs and types of interventions were classified using a classification system previously described in the literature [14]. In brief, DRPs were divided in 17 categories and drug allergy was considered a DRP. Types of interventions were divided in 6 categories. Prior to the start of the study a pilot was conducted on a sample of 30 patients over a 2-week period to assess viability and reliability of the intervention form.

### Clinical significance

Clinical significance of interventions was defined using a scale, developed by Spinewine and colleagues, with 5 categories: 1) minor: no benefit or minor benefit, depending on professional interpretation; 2) moderate: recommendation that brings care to a more acceptable and appropriate level of practice or that might prevent an adverse drug event (ADE) of moderate importance; 3) major: intervention might prevent serious morbidity, including readmission, serious organ dysfunction, serious ADE; 4) extreme: lifesaving; and 5) deleterious: might lead to adverse outcome [14]. Clinical significance of interventions was assessed by an independent panel consisting of one clinical pharmacist and one senior physician. They were from the UK and Australia, with expertise in geriatrics and knowledge of US guidelines (adopted locally). The scale was piloted on a sample of 25 interventions. Written instructions from the pilot were provided to the panel to easy the use of the scale. Panellists had no involvement in the care of patients included in the study. The panel reviewed only interventions accepted by the healthcare team and with direct clinical impact. Significance of each intervention was assessed independently by each panellist. If there was no agreement the panel met to reach consensus on the rating.

### Data analysis

Data were analysed using SPSS software (Statistical Package for the Social Sciences, version 17.0). Characteristics of interventions were assessed using descriptive statistics. Level of agreement for classifying DRPs and types of interventions was assessed during the pilot study. The principal investigator and a clinical pharmacist external to the study coded 33 interventions finding a Cohen’s kappa of 0.80 for underlying DRPs and 0.85 for types of interventions. Agreement was considered substantial and almost perfect respectively [28].

## Results

### Characteristics of participants

Pharmaceutical care was provided to 94 patients. The mean (± SD) age of patients enrolled in the study was 83.3 (± 6.9); 72% were female, 89% were community-dwelling and 16% had ≥1 hospital admission within the previous 6 months. The average length of hospital stay was 8.6 (± 5.4) days. Patients were taking on a regular basis a mean of 4.8 (± 2.0) drugs and the mean number of daily administrations was 6.5 (± 3.3). One administration was defined as the intake of one medicine at a given time during the day (e.g. 1 tablet of X in the morning or 2 tablets of Y in the evening = 1 daily administration) [29].

### Interventions by drug type

A total of 740 interventions were made in 94 patients by the clinical pharmacist. The main drug classes (ATC level 2) involved in interventions were antibacterials for systemic use (J01; 11.1%), anti-parkinson drugs (N04; 10.8%), psycholeptics (N05 – including antipsychotics, anxiolytics, hypnotics and sedatives; 10.3%) and analgesics (N02; 9.6%). A comprehensive list of drug classes involved in interventions is shown in Table 2. Intervention details are shown in Table 3. Of the 740 interventions 690 (93.2%) were fully accepted and 611 (82.6%) were assessed for clinical significance by the independent panel as considered to have an impact on clinical care. Overall, the clinical pharmacist made 63.2% (*n* = 386) interventions of moderate clinical importance and 23.4% (*n* = 143) interventions of major clinical importance. Type of interventions, their acceptance rate and clinical importance are presented in details in Table 4.

**Table 2:** Drug classes involved in interventions (*N* = 740)

Drug Class (ATC level 2)	Interventions *n* (%)	
Antibacterials for systemic use (J01)	82	(11.1)
Anti-parkinson drugs (N04)	80	(10.8)
Psycholeptics (N05)	76	(10.3)
Analgesics (N02)	71	(9.6)
Drugs for acid related disorders (A02)	68	(9.2)
Drugs used in diabetes (A10)	65	(8.8)
Lipid modifying agents (C10)	60	(8.1)
Vitamins (A11)	41	(5.5)
Psychoanaleptics (N06)	38	(5.1)
Beta blocking agents (C07)	33	(4.5)
Antianemic preparations (B03)	19	(2.6)
Diuretics (C03)	18	(2.4)
Antithrombotic agents (B01)	15	(2.0)
Calcium channel blockers (C08)	10	(1.4)
Drugs for obstructive airway diseases (R03)	10	(1.4)
Cardiac therapy (C01)	6	(0.8)
Miscellaneous	48	(6.5)

**Table 3:** Characteristics of interventions (*N* = 740)

Drug-related problem	Interventions *n* (%)		Drugs involved
No specific problem*^,1^	118	(15.9)	antibacterials for systemic use, psycholeptics, drugs used in diabetes, diuretics
Prescription writing error	89	(12.0)	beta blocking agents, anti-parkinson drugs, psycholeptics
Inappropriate follow-up	83	(11.2)	drugs used in diabetes, psychoanaleptics, drugs for acid related disorders, calcium channel blockers
Less costly alternative	74	(10.0)	drugs for acid related disorders, lipid modifying agents, psycholeptics, vitamins
Error in medication history	72	(9.7)	anti-parkinson drugs, analgesics, beta blocking agents, psychoanaleptics
Wrong dose	66	(8.9)	cardiac therapy (digoxin), drugs used in diabetes, vitamins, beta blocking agents
Underuse	54	(7.3)	antianemic preparations, lipid modifying agents
Inappropriate choice of medicine	37	(5.0)	antibacterials for systemic use, antithrombotic agents, psycholeptics
Inappropriate modalities of administration*^,2^	36	(4.9)	antibacterials for systemic use, analgesics
Drug-drug interaction	20	(2.7)	Analgesics
Adverse drug reaction suspected or confirmed*^,3^	16	(2.2)	psycholeptics, psychoanaleptics*^,4^
Duplication	15	(2.0)	drugs for acid related disorders, drugs for obstructive airway diseases
Inappropriate duration of therapy	12	(1.6)	Vitamins
No valid indication	12	(1.6)	Miscellaneous
Modalities of administration not practical for the patient	8	(1.1)	Miscellaneous
Drug-disease interaction (including allergy)	6	(0.8)	Miscellaneous
Other	22	(3.0)	Miscellaneous

*^,1^No underlying drug-related problem; i.e. the clinical pharmacist is asked a drug-related question by a physician in the absence of a drug-related problem regarding a specific patient

*^,2^Modalities of administration refer to frequency of administration, time, route and formulation

*^,3^An adverse drug reaction was defined as a noxious and unintended reaction that occurs at drug doses used in man for prophylaxis, diagnosis or therapy, that could not be linked to another drug-related problem

*^,4^Psychoanaleptics include antidepressants and anti-dementia drugs

**Table 4:** Type, Acceptance Rate and Clinical Significance of Interventions

Intervention Type	*n* (%)	Acceptance Rate (%)			Clinical Significance (%)*^,1^				
		Full	Partial*^,2^	Rejected	Minor	Moderate	Major	Extreme	Deleterious
Educate/inform healthcare professional	151 (20.4)	96.0	2.6	1.3	NA	NA	NA	NA	NA
Change dose	119 (16.1)	94.1	3.4	2.5	1.0	73.7	24.6	1.0	0
Switch to other drug	109 (14.7)	94.5	3.7	1.8	20.2	64.2	14.7	0	0.9
Discontinue drug	105 (14.2)	89.5	6.7	3.8	16.2	59.5	21.6	2.7	0
Add a new drug	86 (11.6)	93.0	4.7	2.3	25.0	44.0	31.0	0	0
Other*^,3^	170 (23.0)	91.8	3.5	4.7	4.3	72.0	23.4	0	0
Total	740 (100)	93.2	3.9	2.8	12.6	63.2	23.4	0.7	0.2

NA = not applicable; i.e. clinical significance not assessed by the independent panel as intervention triggered by a healthcare professional other than the pharmacist and/or intervention not leading to change in drug therapy

*^,1^N = 611 (a total of 611 interventions were assessed for clinical significance by the independent panel; panellists did not assess 129 iinterventions as considered not to impact on clinical care)

*^,2^Advice accepted but partially acted upon

*^,3^Most common intervention types were: monitoring of medications, follow up of medications newly started, advice on form and route of administration of medications and dosage calculations

## Discussion

A total of 143 interventions of major clinical significance were made over a period of 4 months by a clinical pharmacist in an internal medicine ward. That is equivalent to 35 potential major drug-related problems avoided per month. To our knowledge, this is the first study evaluating the effect of a structured communication strategy on acceptance rate of pharmacist interventions. Pharmaceutical care delivered by the clinical pharmacist is likely to have improved appropriateness of prescribing. The majority of interventions made in our study were either of moderate or major clinical significance.

Both the high acceptance of the pharmacist’s interventions (93.2%) and their clinical significance mean that potential harm to patients was avoided and patients care was improved during their stay in the hospital. Other studies using a similar definition of intervention and similar ways of recording them report an acceptance rate of 76%, 88.4% and 87.8% respectively [14, 16, 17]. The acceptance rate of interventions recorded in our study is higher. Driving forces that have contributed for the high acceptance rate of interventions are: aim of the study clearly stated and communicated to healthcare professionals, direct contact with the medical team, nurses and patients, regular presence of the pharmacist on the ward (0.9 full-time equivalent) and pharmacist with appropriate training in clinical pharmacy/pharmacotherapy in the elderly population.

General advice on the use of newer antidiabetics, monitoring of last generation antibiotics and side effects of psycholeptics were frequently asked to the pharmacist. These advices were classified as interventions in the absence of a specific problem (15.9%), were not related to a specific patient and were triggered by physicians highlighting how the pharmacist was fully integrated within the healthcare team.

Collaboration between nurses/medical staff and clinical pharmacists in providing patient care is a relatively new concept in Italy. Currently hospital pharmacists are not ward based and their role is mainly limited to drug dispensing with provision of limited medicine information. This has represented a key challenge emphasizing the need to increase awareness of the new service implemented and build trust with the ward team in order to promote and show potential benefits of pharmaceutical care.

It is expected that the nature and extent of physician-pharmacist collaboration can vary and it can be both episodic and informal rather than as part of a continuum of care. Research looking at interprofessional collaboration between doctors and pharmacists has found that doctors are reluctant to accept the fact that quality and safety of medicine use can improve using the expertise of pharmacists [13]. In our study we have overcome this significant cultural barrier applying models of communication from the commercial world to a healthcare setting in order to foster trust, active collaboration and team working between the two professions with the ultimate aim of enhancing acceptance rate of interventions.

It is expected that lack of awareness and knowledge on the importance of ward based clinical pharmacy services on improving appropriateness of prescribing could be one of the reasons for rejection of pharmacist interventions [30]. Involving all key stakeholders is key in service implementation. The use of communication tools such as the SWOT analysis, SMART goals and the AIDA model are likely to have had a positive impact on the acceptance rate of pharmacist interventions. The SWOT analysis had the aim of explaining to doctors and nurses the aim of the new service implemented and forecast potential issues the team would potentially face in future. Suggestions from stakeholders were considered when planning implementation of the service. This in turn led to involvement of doctors and nurses from conception of the project making them more likely to support it.

The use of SMART goals allowed the breakdown of the aim of the study in more simple and measurable objectives which in turn increased awareness of doctors and nurses on the role of the clinical pharmacist at ward level. The AIDA model helped delivering results of the project throughout implementation of the service in a timely and efficient way increasing involvement of doctors and nurses. Communication was delivered, in practice, via power point presentations on key clinical topics tailored for the specific audience and attending meetings to update stakeholders on progress of the service being implemented. Information and concepts delivered using these communication tools were then reinforced via email 1 week after the presentation/meeting. Content of meetings and presentations was further reminded to healthcare staff using face to face communication on a daily basis. A monthly email was sent to staff summarizing key topics discussed in the last 30 days and asking for feedback.

The average number of medication per patient on admission was lower than reported in the literature [14, 31]. This was due to the fact that doctors withhold several drugs when patients were admitted and reintroduced them usually within the following 72 h when the patient’s clinical picture was better defined. The clinical pharmacist spent an important part of his time providing ward-based teaching to healthcare professionals. This is the reason we observed a high number of interventions (20.4%) aiming to educate the healthcare team. A cohort study found that 25.8% of elderly had at least one potentially inappropriate medication prescribed [5]. Our study found that 14.2% of interventions aimed at discontinuing one or more medication suggesting the clinical pharmacist contributed to avoid unnecessary prescribing and generated cost savings. Cost savings were also generated by the pharmacist advising less costly alternative (10%) drugs. In our study it was found that one of the main drug classes involved in interventions was psycholeptics (10.3%). The use of these drugs is associated with falls in elderly patients [9] suggesting interventions made by the clinical pharmacist in addressing DRPs caused by psycholeptics had the potential to reduce falls. Our study found that 9.7% of interventions were made to address DRPs related to errors in medication history. A US study showed that 24.2% medication history discrepancies resulted in discrepancies during hospitalisation [32] highlighting that interventions addressing medication history DRPs might have reduced these discrepancies.

The way interventions are communicated between doctors and pharmacists affects acceptance rates. Research has shown that written communication in the form of paper notes or entries into the medical records result in lower acceptance rate of 39–70% [17]. High acceptance rate of 88.4% has been reported when face-to-face communication has been used [16]. These findings are confirmed in our study where interventions either partially accepted or rejected were communicated attaching a pharmacist intervention note to the drug chart and interventions accepted were communicated and discussed with doctors orally.

To our knowledge this is the first study reporting on the introduction of a clinical pharmacist on an acute care hospital ward in Italy. The trust has been accredited by The Joint Commission (JC) as academic hospital in 2014 and benefits of the clinical pharmacy service introduced are in line with patient safety goals required by JC [33].

Our study has provided evidence to policy makers that a well thought and tailored communication strategy can have a positive impact on acceptance rate of interventions made by pharmacists. The ward-based clinical pharmacy model implemented could have a significant impact on the improvement of healthcare in hospitals. The service could be implemented within the whole trust using the same model and trained clinical pharmacists should be introduced at ward level. Pharmacoeconomy should be a key component in future developments of clinical pharmacy as cost-effective quality improvements are given priority within healthcare settings [8].

The main strength of the study is that clinical assessment of interventions by an external independent panel made results of the study more reliable. Furthermore the substantial level of agreement when coding DRPs and types of interventions highlights the extent to which the data collected in the study are correct representations of the variable measured. Current research shows that ward pharmacy services like the one described can be successfully implemented in countries having low level of clinical pharmacy input within the hospital setting using the correct communication strategy.

The study had a number of limitations. The service was implemented by a single clinical pharmacist on a 30 beds acute internal medicine unit. A multicentre study was not feasible at the time due to funding issues. The results of the study should be interpreted carefully due to lack of generalizability. However, we believe the clinical pharmacy model described can be replicated elsewhere employing trained staff and the communication strategy described as we would expect other hospitals have a similar operating health care system. The study was relevant in our context as aimed at raising awareness of hospital based clinical pharmacy services amongst policy makers nationwide. Lack of control and the employment of one clinical pharmacist in the study potentially limited interpretation of results. However, we used an external independent panel to assess interventions and findings of the study were interpreted and discussed by the whole research team reducing the risk of bias. Previous work has shown that clinical pharmacists providing pharmaceutical care to inpatients might improve their health-related quality of life [34] however this was not measured in our study. Persistence of interventions after discharge was not assessed due to lack of time. As clinical pharmacy is patient oriented, patients’ reported experience of pharmaceutical care should have been measured using a patient satisfaction questionnaire.

## Conclusions

We believe the communication strategy adopted has increased both trust and collaboration between doctors and the pharmacist and this has had in turn a positive impact on acceptance rate of interventions. Pharmaceutical care delivered by the clinical pharmacist is likely to have had beneficial outcomes. Clinical pharmacy services like the one described should be implemented widely to increase patient safety. We reckon hospital pharmacy stakeholders should be more proactive and invest in ward-based clinical pharmacy services as the impact on patient safety is likely to be immediate. Introduction of the service is justified by the need to improve appropriateness of prescribing. Clinical pharmacy services like the one described must be evidence based and employ staff with appropriate training.

## Abbreviations

ADE: Adverse drug event
ADR: Adverse drug reaction
AIDA: Awareness, interest, desire, action
ATC: Anatomical therapeutic chemical
DRP: Drug-related problem
JC: Joint Commission
PCT: Primary care team
SMART: Specific, Measurable, Achievable, Realistic and Timely
SPSS software: Statistical Package for the Social Sciences
SWOT: Strengths, Weaknesses, Opportunities and Threats
